# Effects of regularisation priors on dynamic PET Data

**DOI:** 10.1186/2197-7364-1-S1-A46

**Published:** 2014-07-29

**Authors:** Liliana Caldeira, Juergen Scheins, Nuno da Silva, Michaela Gaens, N Jon Shah

**Affiliations:** Institute of Neuroscience and Medicine-4, Forschungszentrum Juelich, Kragujevac, Germany

Dynamic PET provides temporal information about tracer uptake. However, each PET frame has usually low statistics, resulting in noisy images. The goal is to study effects of prior regularisation on dynamic PET data. Quantification and noise in image-domain and time-domain as well as impact on parametric images is assessed.

Dynamic PET data for the Siemens 3T MR-BrainPET was simulated with time-activity curves (TAC) of [1] F-FDG obtained analytically using realistic values for the kinetic model (Table 1). The total number of true counts was 6x10^8^ and scatter and random fractions were both 35%. The data consists of 23 frames as applied in our clinical protocol. For reconstruction, the Ordinary Poisson Ordered Subset Expectation Maximisation (OP-OSEM) method was used in PRESTO [1], which allows to use several 3D priors [2]. The Median Root Prior (MRP) used a 3x3x3 neighbourhood and a Bayes parameter of 0.1 for all frames. Patlak parametric images were calculated using PMOD software.

**Table 1:** Kinetic values used for simulation of two tissues: White-Matter (WM) and Gray-Matter (GM), extracted from real acquired volunteer data.

	k1 (mL/cc/min)	k2 (min-^1^)	k3 (min^-1^)	k4 (min^-1^)
White-Matter (WM)	0.054	0.111	0.0045	0.0059
Gray-Matter (GM)	0.103	0.133	0.063	0.0068

**Figure 1:**
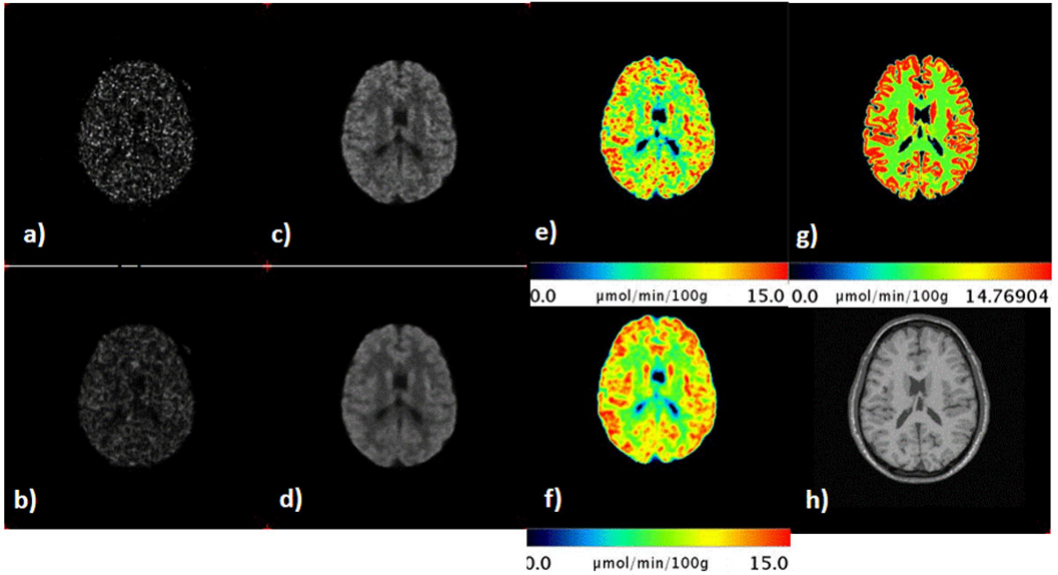
a) Reconstructed images without MRP corresponding to Frame 14 (2.77x10^6^ counts); b) Reconstructed images with MRP corresponding to Frame 14 (2.77x10^6^ counts); c) Reconstructed images without MRP corresponding to Frame 24 (119x10^6^ counts); d) Reconstructed images with MRP corresponding to Frame 24 (119x10^6^ counts); e) Patlak parametric image of reconstructed images without prior; f) Patlak parametric image of reconstructed images with prior; g) Patlak Parametric Images of ground truth; h) MRI image for anatomical reference.

Figure 1 shows that the MRP OP-OSEM reduces image noise in WM, GM and AIF: from 50% reduction in low-count frames to 10% reduction in high-count frames (e.g. Frame 24). Furthermore, Patlak parametric images also look smoother for MRP OP-OSEM than for OP-OSEM.

**Figure 2:**
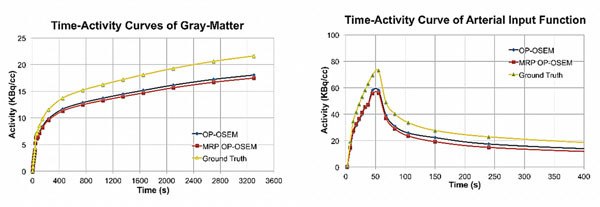
TAC using OP-OSEM reconstruction without and with MRP for two different regions: a) GM and b) AIF.

Figure 2 shows similar quantification for both reconstruction methods (with similar RCs). OP-OSEM presents higher RCs than MRP OP-OSEM in high-count frames (up to 9%), while in low-count frames MRP OP-OSEM presents higher RCs (up to 9%). This is probably due to the fact that the Bayes parameter is count-dependent.

This study shows improvement on PET image quality in terms of noise (up to 50% reduction) as well as in parametric images when using prior regularisation in dynamic PET data. Both OP-OSEM and MRP OP-OSEM show similar quantification, with higher RCs for MRP OP-OSEM in low-count frames.
